# Is CT Angiogram of the Abdominal Vessels Needed following the Diagnosis of Ischemic Colitis? A Multicenter Community Study

**DOI:** 10.1155/2014/756926

**Published:** 2014-02-12

**Authors:** Muhammed Sherid, Salih Samo, Samian Sulaiman, Husein Husein, Sankara N. Sethuraman, John A. Vainder

**Affiliations:** ^1^Division of Gastroenterology, Department of Internal Medicine, CGH Medical Center, 100 East Le Fevre Road, Sterling, IL 61081, USA; ^2^Division of Gastroenterology, Department of Internal Medicine, Saint Francis Hospital, 355 Ridge Avenue, Evanston, IL 60202, USA; ^3^Division of Gastroenterology, Department of Internal Medicine, University of Tishreen, Aleppo Street, P.O. Box 2230, Latakia, Syria; ^4^Department of Mathematics and Computer Science, Augusta State University, 2500 Walton Way, Augusta, GA 30904, USA

## Abstract

*Background*. CT angiogram is frequently obtained after diagnosis of ischemic colitis (IC). *Aims*. To investigate the vascular findings of CT angiogram as compared to contrast-enhanced CT scan and whether this modality changes the management or prognosis of IC. *Methods*. We conducted a retrospective analysis of patients with IC from 2007 to 2013. *Results*. CT angiogram was performed in 34 patients (28.81%), whereas contrast-enhanced CT scan was performed in 54 patients (45.76%). In CT angiogram group, 8 patients (23.5%) had atherosclerotic changes. Stenosis was found in 12 patients (35.3%) (9: celiac trunk, 3: SMA). Among this group, one patient underwent colectomy and another underwent angioplasty of the celiac trunk who died within 30 days. Among contrast-enhanced CT scan group, 5 patients (9.3%) had atherosclerotic changes. Stenosis was found in 5 patients (9.3%) (3: celiac trunk, 1: SMA, and 1: IMA). Among this group, 3 patients had colectomy and one died within 30 days. There was no statistical difference between both groups in all vascular findings except the stenosis which was higher in CT angiogram group (*P* = 0.0025). Neither the need for surgery nor all-cause mortality was different between both groups. *Conclusion*. CT angiogram did not provide any useful findings that altered the management or the prognosis of IC.

## 1. Introduction

Blood circulation of the gastrointestinal (GI) tract consists of three major splanchnic arteries which supply the stomach to the rectum: celiac trunk, superior mesenteric artery (SMA), and inferior mesenteric artery (IMA). The colon is protected against ischemia by a rich collateral blood circulation; however, these collateral networks are considered relatively limited as compared to those in the stomach and the small bowel. The two main arteries supplying the colon are the SMA and the IMA. The vascular anatomy of colonic vessels is highly variable between individuals, exposing the colon to ischemia especially in the watershed areas and the right colon [1]. Splenic flexure (Griffiths' point) and rectosigmoid junction (Sudeck's point) are known as watershed areas because of the critical points of blood flow between two different vascular supplies: the SMA and the IMA and the IMA and the internal iliac artery, respectively [1–5]. The marginal vessel is poorly developed in the right colon in up to 50% of people, making the right colon susceptible to ischemic colitis [1].

Mesenteric (bowel) ischemia is classified into three entities which vary in their etiologies, presentation, management, and prognosis: ischemic colitis, acute mesenteric (bowel) ischemia, and chronic mesenteric (bowel) ischemia. Ischemic colitis is a consequence of inadequate blood flow to the colon, which returns to normal quickly. Ischemic colitis is usually self-limited disease that resolves completely with conservative treatment. Although it can occur in young persons, ischemic colitis is known as an elderly disease. It is multifactorial and the precipitating factors are not identifiable in many cases. It usually occurs without occlusion in major blood vessels.

Acute mesenteric ischemia refers to the sudden onset of acute intestinal hypoperfusion. It presents usually with severe acute abdominal pain that is out of proportion to tenderness on physical examination. It is usually due to acute thromboembolic events in one or more of the mesenteric vessels that lead to acute arterial occlusion [6, 7].

Chronic mesenteric ischemia refers to slowly progressive intestinal angina that is characterized by chronic episodic postprandial abdominal pain, leading to fear of eating (sitophobia) and weight loss which occurs typically in elderly women [7–9]. It is usually due to progressive multivessel mesenteric atherosclerosis that leads to chronic critical arterial occlusion [7].

The role of computed tomography (CT) angiogram in both acute and chronic mesenteric ischemia is well established; however, its role in ischemic colitis is uncertain. CT angiogram is frequently performed after the diagnosis of ischemic colitis looking for potential treatable vascular abnormalities. The aim of our study was to investigate the vascular radiological findings of CT angiogram as compared to the usual contrast-enhanced CT scan and to investigate whether this modality changes the management or prognosis of ischemic colitis.

## 2. Methods

We conducted a retrospective analysis of all patients with the diagnosis of ischemic colitis from January 2007 to January 2013. The study was undertaken in two different community hospitals (CGH Medical Center in Sterling, IL, and Saint Francis Hospital in Evanston, IL) after obtaining the Institutional Review Board (IRB) approval from each institution.

We systematically reviewed the medical records for the following data: demographic details, clinical signs and symptoms, laboratory studies, CT angiogram and CT scan findings, endoscopic and histologic features, anatomic location of ischemic colitis, comorbidities, concomitant use of medications, need for surgical intervention, blood transfusion, hospital stay, requirement for intensive care unit (ICU) and mechanical ventilation, and all-cause mortality within 30 days.

The aim of our study was to investigate the radiologic vascular findings of CT angiogram compared to the contrast-enhanced CT scan and to investigate whether this modality changes the management or prognosis of ischemic colitis. Cases of ischemic colitis were identified by using the International Classification of Diseases-ninth version (ICD-9) codes (the code 557.0: acute vascular insufficiency and the code 557.9: unspecific vascular insufficiency) because there are no specific codes for ischemic colitis. All cases were carefully audited individually by chart review to determine the diagnosis of ischemic colitis. The diagnosis of ischemic colitis was made based on clinical signs and symptoms that were consistent with ischemic colitis with negative stool studies for infections, with at least one diagnostic study that was consistent with ischemic colitis (CT scan, colonoscopy, or histopathology).

Exclusion criteria were age <18 years, pregnancy, positive studies for enteric pathogens, colonic ischemia due to trauma or mechanical causes (bowel obstruction, volvulus, hernia, etc.), acute mesenteric ischemia, chronic bowel ischemia, acute flare of inflammatory bowel disease, and radiological or colonoscopic evidence of diverticulitis. In addition, we excluded any case with equivocal or uncertain diagnosis of ischemic colitis or where ischemic colitis was merely considered in the deferential diagnosis but never confirmed with objective modalities.

*Statistical Analysis*. All patients' information was entered into a Microsoft Excel spreadsheet in a coded format which was locked with a password. The data were analyzed using the SAS software (SAS Institute Inc., Cary, NC). Frequency distribution indicators were calculated by descriptive analysis. The results for quantitative variables were reported as mean and standard variation. A chi-square analysis, Fisher's exact test, and Pearson's correlation were conducted. A 2-sided *P* value of <0.05 was considered statistically significant.

## 3. The Results

### 3.1. Patients' Clinical Characteristics (Table 1)

A total of 118 patients with ischemic colitis were identified from January 2007 to January 2013. The mean age was 69.41 ± 15.07 years with a vast majority of being white females (>80%). Ischemic colitis occurred in 15 patients (12.7%) younger than 50 years old. The mean body mass index (BMI) was 28.1 ± 6.6. Abdominal pain was the most common presenting symptom in 82.2%, followed by rectal bleeding in 74.6%, and then diarrhea in 55.1%. Nausea, vomiting, fever, abdominal distension, and peritoneal signs were less frequently seen. The median interval from the onset of symptoms to seeking medical attention was 17.0 hours.

The most common comorbidities associated with ischemic colitis were hypertension (HTN), hyperlipidemia (HLD), coronary artery disease (CAD), diabetes mellitus (DM), and atrial fibrillation (AF) (79.5%, 58.1%, 31.6%, 21.4%, and 18%, resp.). History of any abdominal surgeries was present in 56.9%, hysterectomy in 31%, cholecystectomy in 25%, and appendectomy in 19%. The most concomitant medications used were beta-blockers, angiotensin converting enzyme inhibitors (ACEIs), statins, aspirin, and calcium channel blockers (48.3%, 46.6%, 45.7%, 45.7%, and 33.6%, resp.).

The mean length of hospital stay was 6.7 ± 7.6 days. We used the website http://www.timeanddate.com/ to calculate the length of hospital stay and we included both admission and discharge days. We did not calculate the length of stay hourly. The need for blood transfusion, ICU stay, mechanical ventilation, and surgery was in 20.3%, 19.5%, 11.9%, and 11.9%, respectively. In 9.3% of cases, ischemic colitis developed while patients were in the hospital for different reasons. Ischemic colitis recurred in 8.5% of cases during the 6-year study period. Death within 30 days of the diagnosis of ischemic colitis occurred in 5 patients (4.2%) of the total 118 patients with different causes of death (ischemic colitis [2], sepsis [2], and sudden cardiac death [1]). The mean interval from the admission to death was 19.8 ± 10.3 days (3, 18, 22, 27, and 29 days). Severe ischemic colitis, which was defined as ischemic colitis that required surgery or resulted in death within 30 days of diagnosis, occurred in 12.7%.

Identifiable direct predisposing factors of ischemic colitis were constipation in 13.6%, hypotension in 5.9%, and drugs and vasculitis—as one group—in 5.1%.

### 3.2. Diagnostic Studies (Table 2)

The mean white blood cells (WBC), hemoglobin (Hb), albumin, bicarbonate, sodium (Na), creatinine, alanine aminotransferase (ALT), amylase, lipase, glucose, and lactic acid on admission were 12.96 ± 6.23, 13.04 ± 1.89, 3.63 ± 0.53, 25.32 ± 3.70, 137.20 ± 13.78, 1.38 ± 0.92, 29.92 ± 16.82, 104.71 ± 173.09, 118.96 ± 116.86, 135.95 ± 71.10, and 4.54 ± 11.69, respectively. Colonic radiological findings were available in 76.3% with wall thickening, pericolonic fat stranding, and induration being the most common findings (72.2%, 60%, and 22.2%, resp.). Colonoscopy was performed in 75.4% with erythema, edema, and erosions/ulcerations being the most three common findings (64.1%, 58.4%, and 52.8%, resp.). Histopathology (either from endoscopic biopsy or surgery) was available in 79.7% with acute inflammation, necrosis/exudate, and chronic inflammation being the most common histologic findings (72.3%, 41.5%, and 35.1%, resp.).

The anatomic location of the involved colonic segments was based on surgery report, CT scan, and colonoscopy findings. When surgery was performed, surgical findings were taken for involved location regardless of the colonoscopy and radiology findings. When surgery was not performed and there was discrepancy between CT scan and colonoscopy, the colonoscopy findings were taken. Location of ischemic colitis was divided into right colon and left colon and then to specific segments of the colon (rectum, rectosigmoid junction, sigmoid, descending colon, splenic flexure, transverse colon, hepatic flexure, ascending colon, and cecum). Some patients had more than one segment affected. The left colon was affected in 84.8%, whereas the right colon was affected in 14.3% of cases. There was one case of pancolitis (0.9%). The descending colon was the most common affected segment (65.2%), followed by the splenic flexure (51.8%), and then the sigmoid colon (43.8%). The rectum was affected only in 2.7%.

### 3.3. Vascular Radiologic Findings (Table 3)

CT angiogram was performed in 34 patients (28.81%) (CT angiogram group), whereas the contrast-enhanced CT scan was performed in 54 patients (45.76%) (the contrast-enhanced CT scan group), and the CT scan without contrast was performed in 31 patients (26.27%). We compared the vascular radiological findings of CT angiogram group to the contrast-enhanced CT scan group which matched in terms of age and gender. Some patients had only one modality whereas others had both modalities. Patients who had both modalities were included in both groups. In the CT angiogram group, 8 patients (23.5%) had atherosclerotic changes (7 cases in the celiac trunk, 5 in the SMA, and 2 in the IMA), of which 2 cases had 2 arteries affected and 2 cases had 3 arteries affected. Stenosis was found in 12 patients (35.3%) (3 cases of mild stenosis (30–50%), 7 moderate (50–70%), 1 case of severe stenosis (>70%), and 1 case of total occlusion). The stenosis was located in the celiac trunk in 9 patients including the severe case and the total occlusion case and in the SMA in 3 patients. There was no case of stenosis in the IMA. The stenosis was in the origin of the arteries in 6 patients and in the proximal portion in the other half. The IMA was reported as not visualized in 4 patients. Abdominal aortic aneurysm (AAA) was found in 3 cases (8.8%). Among all patients who had CT angiogram, only one patient, who did not have any arterial stenosis or atherosclerotic changes, underwent colectomy. Only the patient with severe stenosis in the celiac trunk underwent angioplasty of the celiac trunk and died on the 29th day of hospitalization.

Among the contrast-enhanced CT scan group, 5 patients (9.3%) had atherosclerotic changes (4 cases in the celiac trunk, 4 in the SMA, and 1 case in the IMA), of which 2 cases had 2 arteries involved and one case had all three vessels involved. Stenosis without classification was found in 5 patients (9.3%) and was located in the origin of the arteries (3 in the celiac trunk, 1 in the SMA, and 1 in the IMA). The IMA was not seen in four cases whereas the celiac trunk was not seen in one case. AAA was found in 6 cases (11.1%). Among the contrast-enhanced CT scan group, 3 patients had colectomy and another one died within 30 days of hospitalization. There was no statistically significant difference between both groups in all vascular radiological findings except the stenosis which was higher in CT angiogram group (*P* = 0.0025). Neither the need for surgery nor all-cause death within 30 days of the diagnosis of ischemic colitis was different between both groups. There were no cases of mesenteric venous abnormalities or thromboembolic events detected in either CT angiogram or the CT scan.

## 4. Discussion

The incidence of ischemic colitis in general population ranges from 4.5 to 44 cases per 100,000 person-years [10]. It accounts for 8.7–18% of cases of acute lower GI bleeding; however, in some studies it is as high as 23.7% of cases [11–13]. Ischemic colitis is the most common form of ischemia in the GI tract. It is an elderly disease with the mean age of 69.4 years in our study, which is in accordance with previous studies [10, 14]. It mainly affects people older than 65 years; however, 12.7% in our group were younger than 50 years. Younger persons account for 10–15% of all cases of ischemic colitis; however, it has been described as high as 34% [15–17]. The most common presenting symptoms are abdominal pain, rectal bleeding, and diarrhea [14, 18, 19]. Cardiovascular disease and cardiovascular risk factors such as HTN, HLD, and DM are the most common comorbidities associated with ischemic colitis which have been repeatedly documented in studies including our study [1–5, 12, 14, 18–23]. Severe ischemic colitis (that required surgery or resulted in death within 30 days) occurred in 12.7% of our patients which is congruent with the recent largest prospective multicenter study by Montoro and his colleagues [19]. However, the rate of severe ischemic colitis varies widely among studies and ranges from 9.1 to 34%, but it has been reported as high as 45% [2, 14, 18, 20, 24–27]. Colonoscopy and CT scan are able to establish the diagnosis of ischemic colitis along with clinical scenarios in more than 95% of cases; the rest is diagnosed during emergent/urgent surgery in severe cases [20].

Radiologic findings in ischemic colitis are divided into changes in the bowel wall and its surroundings and changes in the mesenteric vessels. The most common nonvascular radiologic findings in ischemic colitis are colonic wall thickening and pericolonic fat stranding [22, 24, 28, 29]. The mean colonic wall thickness in ischemic colitis is 8-9 mm which ranges from 2 to 20 mm [22, 30]. Ischemic colitis is usually segmental which is also demonstrated on CT scan with a mean length of 19 cm [7, 22, 28]. The colonic wall thickening is usually circumferential with a homogeneous or heterogeneous appearance, depending on the degree of edema, inflammation, and bleeding [7, 22, 28]. Ischemic colitis is the most common cause of colonic wall thickening detected on CT scan in patients admitted for abdominal pain [31]. Intramural, intravascular, and intraperitoneal free air are less frequent findings but are considered ominous findings that are associated with severe cases that lead to either surgery or death [7, 22, 28].

Although the main purpose of the contrast-enhanced abdominal CT scan is the evaluation of bowel structures and other organs and not the anatomical details of visceral arteries, the contrast-enhanced CT scan has an advantage of detecting some abnormalities in the mesenteric vasculature for atherosclerosis or thromboembolic occlusion [9]. CT angiogram refers to a multidetector row CT that combines multiple rows of detectors and a faster rotation with narrow collimation [9, 32]. This technique allows reconstruction of the images to create three-dimensional pictures that is comparable to the conventional angiogram. In many cases, this modality has eliminated the need for further imaging studies such as Doppler ultrasonography or conventional angiogram. The sensitivity and specificity of CT angiogram in detecting acute mesenteric ischemia are 96% and 94%, respectively [33].

The mechanisms of ischemic colitis include transient colonic hypoperfusion, local vasoconstriction, mesenteric thromboembolism, and vasculitis [3–5]. CT angiogram is frequently performed following the diagnosis of ischemic colitis to look for the etiology of ischemic colitis. The rate of performing CT angiogram in previous studies is not well documented, but it has been ranged from 0 to 5.8% [9, 34]. However, the usefulness of CT angiogram after diagnosis of ischemic colitis has not been addressed in clinical trials.

In our study, CT angiogram was performed in 28.8% of patients, but it did not provide further useful findings than the contrast-enhanced CT scan as all vascular radiological findings did not differ between both groups except for mesenteric stenosis. It is expected that CT angiogram will detect more stenosis than the contrast-enhanced CT scan as the primary goal for the former is to detect vascular abnormalities and classify the stenosis. Nonetheless, mesenteric arterial stenosis is a frequent incidental finding, especially in older patients with cardiovascular risk factors. In autopsy studies, mesenteric arterial stenosis has been found in 6% in people younger than 40 years, in 14% in those with age of 40–59 years, and in 67% in those over 80 years [8]. Furthermore, 40% of patients who were admitted for various clinical reasons excluding acute mesenteric ischemia had stenosis in one of the mesenteric arteries detected on CT angiogram and 15.3% had multivessel disease in a study of 85 patients by Cardin and his colleagues [35]. In a study of 129 patients with ischemic colitis, mesenteric angiographic studies were performed in 11 patients (8.5%), which detected the IMA occlusion in only one patient (9.1%) [23]. In another study of 54 patients with ischemic colitis, 6 patients (11.1%) had angiographic studies which were normal except in one case (16.7%) which showed severe stenosis in the IMA associated with arteriosclerotic disease [22]. In a third study of 97 patients of ischemic colitis, angiography was performed in 9 patients (9.3%) and revealed a stenosis in the SMA in only one patient (11.1%) [29]. It was not documented in these three studies whether the angiographic studies were conventional angiogram, CT angiogram, or both.

It has been shown that mesenteric atherosclerosis in surgical specimens is associated with worse survival and is an independent factor for poor long-term survival [36]. Mesenteric atherosclerosis probably reflects systemic arterial atherosclerosis as most patients with ischemic colitis who expire in long-term followup die from cardiovascular diseases and it is judicious to treat those who survive from ischemic colitis with aggressive secondary prevention measures and should be considered as high-risk cardiovascular patients [36].

Moreover, CT angiogram did not alter the management and the prognosis of ischemic colitis in our study as the need for surgery (colectomy, vascular surgery, or angioplasty) or death within 30 days was not different between both groups. Although it has been recommended by many authors against the routine use of CT angiogram following the diagnosis of ischemic colitis, this is the first study that supports their recommendations [2, 20, 37]. In addition to not providing further information than the contrast-enhanced CT scan, CT angiogram has many disadvantages including increased exposure to radiation, potential nephrotoxicity due to the contrast use, and additional costs.

Limitations of our study include the small sample of patients and the retrospective design of the study, which carries some inherent errors such as the deficiency in chart documentation. However, it is the first study that addressed the radiological findings of CT angiogram as compared to the contrast-enhanced CT scan and its effects in the management and the prognosis of ischemic colitis.

In conclusion, CT angiogram was performed in more than a quarter of our patients following the diagnosis of ischemic colitis. CT angiogram detected more stenosis than the contrast-enhanced CT scan as the primary goal of performing the latter is not for detecting this finding. However, CT angiogram did not change the management or the prognosis of ischemic colitis. We recommend against the routine use of CT angiogram after the diagnosis of ischemic colitis. CT angiogram might be considered in more severe cases of ischemic colitis especially with right colon involvement or in instances of the need for excluding acute mesenteric ischemia based on clinical scenarios.

## Figures and Tables

**Table 1:** Clinical characteristics of both groups.

	Ischemic colitis (*N* = 118)
Mean age (years)	69.41 ± 15.07
Gender	
Female	98 (83.1%)
Male	20 (16.9%)
Age group	
<50 years	15 (12.7%)
≥50 years	103 (87.3%)
Race group	
White	97 (82.2%)
Others	21 (17.8%)
Mean BMI ± SD	28.09 ± 6.63
Smoking habits	
Never smoked	68 (58.1%)
Ex-smoker	24 (20.5%)
Current smoker	25 (21.4%)
Missing data	1
Clinical symptoms/signs	
Abdominal pain	97 (82.2%)
Nausea	43 (36.4%)
Vomiting	34 (28.8%)
Diarrhea	65 (55.1%)
Rectal bleeding	88 (74.6%)
Abdominal distension	8 (5.8%)
Fever	18 (15.3%)
Peritoneal signs	6 (5.1%)
Mean SBP ± SD	135.06 ± 33.21
Mean DSP ± SD	70.51 ± 15.68
Mean HR ± SD	83.43 ± 21.53
Comorbidities	
HTN	93 (79.5%)
HLD	68 (58.1%)
CAD	37 (31.6%)
DM	25 (21.4%)
CHF	10 (8.6%)
AF	21 (18%)
PVD	11 (9.4%)
CVA	13 (11.1%)
COPD	17 (14.5%)
CKD	15 (12.8%)
DVT	4 (3.4%)
IBS	3 (2.6%)
AAA	9 (7.7%)
Autoimmune diseases	8 (6.8%)
Missing data	1
Abdominal surgery (any)	66 (56.9%)
Appendectomy	22 (19%)
Cholecystectomy	29 (25%)
Hysterectomy	36 (31%)
Missing data	2
Drugs	
Clopidogrel (Plavix)	24 (20.7%)
Aspirin	53 (45.7%)
Statins	53 (45.7%)
Calcium channel blockers	39 (33.6%)
*β*-Blockers	56 (48.3%)
ACEIs	54 (46.6%)
ARBs	13 (11.2%)
Diuretics	34 (29.3%)
NSAIDs	11 (9.5%)
Digoxin	6 (5.2%)
Warfarin	14 (12.1%)
Antidepressants/antipsychotics	34 (29.3%)
Missing data	2
Mean hospital stay ± SD (days)	6.67 ± 7.62
ICU stay (percentage)	23 (19.5%)
Mechanical ventilation	14 (11.9%)
IC event occurred during hospitalization	11 (9.3%)
Recurrence	10 (8.5%)
Blood transfusion	24 (20.3%)
Surgery	14 (11.9%)
Death in 30 days	5 (4.2%)
Severe ischemic colitis (required surgery or died)	15 (12.7%)
Direct causes	
Constipation	16 (13.6%)
Hypotension	7 (5.9%)
Drug/vasculitis	6 (5.1%)

AAA: abdominal aortic aneurysm, ACEIs: angiotensin converting enzyme inhibitors, ARBs: angiotensin receptor blockers, AF: atrial fibrillation, BMI: body mass index, CAD: coronary artery disease, CHF: congestive heart failure, CKD: chronic kidney disease, COPD: chronic obstructive pulmonary disease, CVA: cerebrovascular disease, DM: diabetes mellitus, DVT: deep venous thrombosis, HLD: hyperlipidemia, HTN: hypertension, IBS: irritable bowel syndrome, IC: ischemic colitis, ICU: intensive care unit, NSAIDs: nonsteroidal anti-inflammatory drugs, and PVD: peripheral vascular disease.

**Table 2:** Laboratory, radiology, colonoscopy, and histopathology findings between both groups.

	Ischemic colitis (*N* = 118)
Mean WBC ± SD at admission	12.96 ± 6.23
Mean highest WBC during hospital stay ± SD	14.49 ± 6.84
Mean Hb ± SD	13.04 ± 1.89
Mean lowest Hb during hospital stay ± SD	10.38 ± 2.07
Mean albumin ± SD	3.63 ± 0.53
Mean bicarbonate ± SD	25.32 ± 3.70
Mean Na ± SD	137.20 ± 13.78
Mean Cr ± SD	1.38 ± 0.92
Mean ALT ± SD	29.92 ± 16.82
Mean amylase ± SD	104.71 ± 173.09
Mean lipase ± SD	118.96 ± 116.86
Mean glucose ± SD	135.95 ± 71.10
Mean lactic acid ± SD	4.54 ± 11.69
CT scan findings	
Available	90 (76.3%)
Normal CT	10 (11.1%)
Wall thickening	65 (72.2%)
Induration	20 (22.2%)
Pericolonic fat stranding	54 (60%)
Loss of haustra	5 (5.6%)
Free intra-abdominal fluid	16 (17.8%)
Pneumatosis coli	7 (7.8%)
Portal/mesenteric venous gas	4 (4.4%)
Pneumoperitoneum	4 (4.4%)
Bowel dilation	13 (14.4%)
Colonoscopy findings	
Performed	89 (75.4%)
Edematous mucosa	52 (58.4%)
Erythema	57 (64.1%)
Erosions/ulcerations	47 (52.8%)
Friability/active bleeding	24 (25%)
Exudate/necrosis	9 (10.1%)
Stricture	2 (2.3%)
Missing data	1
Histology findings	
Available	94 (79.7%)
Normal histology	4 (4.3%)
Edema	8 (8.5%)
Epithelium loss/ulceration	31 (33%)
Crypt loss	8 (8.5%)
Acute inflammation	68 (72.3%)
Chronic inflammation	33 (35.1%)
Capillary thrombosis	5 (5.3%)
Necrosis/exudate	39 (41.5%)
Submucosal hemorrhage	20 (21.3%)
Vascular congestion	5 (5.3%)
Mucosal/transmural infarct	7 (7.5%)
Chronic ulcer	9 (9.6%)
Location	
Left colon	95 (84.8%)
Right colon	16 (14.3%)
Pancolitis	1 (0.9%)
Rectum	3 (2.7%)
Rectosigmoid junction	14 (12.5%)
Sigmoid	49 (43.8%)
Descending colon	73 (65.2%)
Splenic flexure	58 (51.8%)
Transverse colon	33 (29.5%)
Hepatic flexure	8 (7.1%)
Ascending colon	13 (11.6%)
Cecum	12 (10.7%)
Missing data	6

ALT: alanine aminotransferase, Cr: creatinine, CT: computed tomography, Hb: hemoglobin, Na: sodium, and WBC: white blood cells.

**Table 3:** Vascular radiological findings between both groups.

	CT angiogram (*N* = 34 patients)	Contrast-enhanced CT scan (*N* = 54 patients)	*P* value
Average age	63.85	65.74	NS
Gender			0.2155
Female	30 (88.2%)	42 (77.8%)	
Male	4 (11.8%)	12 (22.2%)	
Atherosclerotic changes	8 (23.5%)	5 (9.3%)	0.066
Stenosis	**12 (35.3%)**	**5 (9.3)**	**0.0025**
Celiac trunk not seen	0 (0%)	1 (1.9%)	0.425
SMA not seen	0 (0%)	0 (0%)	NS
IMA not seen	4 (11.8%)	4 (7.4%)	0.4887
AAA	3 (8.8%)	6 (11.1)	0.73
Surgery (colectomy)	1 (2.9%)	3 (5.6%)	0.566
Vascular surgery or angioplasty	1 (2.9%)	0 (0%)	0.205
Death	1 (2.9%)	1 (1.9%)	0.738

AAA: abdominal aortic aneurysm, IMA: inferior mesenteric artery, NS: non-significance, SMA: superior mesenteric artery, and Bold values: statistically significant.
